# Feasibility of melting fingerprint obtained from ISSR-HRM curves for marine mammal species identification

**DOI:** 10.7717/peerj.11689

**Published:** 2021-06-25

**Authors:** Wannapimol Kriangwanich, Kittisak Buddhachat, Anocha Poommouang, Siriwadee Chomdej, Chatchote Thitaram, Patcharaporn Kaewmong, Kongkiat Kittiwattanawong, Korakot Nganvongpanit

**Affiliations:** 1 Department of Veterinary Biosciences and Public Health, Faculty of Veterinary Medicine, Chiang Mai University, Chiang Mai, Thailand; 2 Excellence Center in Veterinary Bioscience, Chiang Mai University, Chiang Mai, Thailand; 3 Department of Biology, Faculty of Science, Naresuan University, Phitsanulok, Thailand; 4 Department of Biology, Faculty of Science, Chiang Mai University, Chiang Mai, Thailand; 5 Center of Elephant and Wildlife Health, Faculty of Veterinary Medicine, Chiang Mai University, Chiang Mai, Thailand; 6 Phuket Marine Biological Center, Phuket, Thailand

**Keywords:** Dolphin, Dugong, Melting temperature, ISSR

## Abstract

Currently, species identification of stranded marine mammals mostly relies on morphological features, which has inherent challenges. The use of genetic information for marine mammal species identification remains limited, therefore, new approaches that can contribute to a better monitoring of stranded species are needed. In that context, the ISSR-HRM method we have proposed offers a new approach for marine mammal species identification. Consequently, new approaches need to be developed to identify individuals at the species level. Eight primers of the ISSR markers were chosen for HRM analysis resulting in ranges of accuracy of 56.78–75.50% and 52.14–75.93% in terms of precision, while a degree of sensitivity of more than 80% was recorded when each single primer was used. The ISSR-HRM primer combinations revealed a success rate of 100% in terms of discrimination for all marine mammals included in this study. Furthermore, ISSR-HRM analysis was successfully employed in determining marine mammal discrimination among varying marine mammal species. Thus, ISSR-HRM analysis could serve as an effective alternative tool in the species identification process. This option would offer researchers a heightened level of convenience in terms of its performance and success rate. It would also offer field practice to veterinarians, biologists and other field-related people a greater degree of ease with which they could interpret results when effectively classifying stranded marine mammals. However, further studies with more samples and with a broader geographical scope will be required involving distinct populations to account for the high degree of intraspecific variability in cetaceans and to demonstrate the range of applications of this approach.

## Introduction

Many marine mammal species face direct or indirect threats from humans. This has contributed to the deterioration of many marine environmental habitats. A current loss of biological diversity and marine habitats has resulted from various anthropogenic activities such as those associated with the transportation industry, private fishing and hunting activities, and the commercial use of gill nets (Avila, Kaschner & Dormann, 2018; Gales, Hindell & Kirkwood, 2003; Helm et al., 2015; International Union for Conservation of Nature & Natural Resources - IUCN, 2020; Pompa, Ehrlich & Ceballos, 2011; Reeves, McClellan & Werner, 2013; Reeves et al., 2003). According to known disjunct populations and inferences from related cetacean species, it is likely that a large number of cetaceans have been poorly studied. Furthermore, the broad and remote distributions of most cetaceans have made sampling both a challenging and expensive endeavor. Also, the large body size of many marine mammals could severely limit the ability to establish osteological collections for morphological analysis (Taylor et al., 2017). A lack of basic information on cetaceans concerning their ecological function in aquatic ecosystems can affect the conservation status of taxa, putting species or populations at some level of risk for potential long-term or short-term extinction, and could subsequently add to the difficulties associated with the act of conservation planning (Siciliano et al., 2012; Silva et al., 2021). Currently, several marine mammals have been classified as being globally endangered or threatened (International Union for Conservation of Nature & Natural Resources - IUCN, 2020). Marine mammals in Thailand are protected under the Wild Animal Reservation and Protection Act, B.E. 2535. Over the last two decades (2003–2018), there have been 4,539 recorded incidences of stranded marine animals including 1,937 whales and dolphins. This would account for 43% of all stranded marine animals. A primary cause of the increased stranding rates would likely be respiratory infection that can primarily be attributed to an increase in plastic pollution (Department of Marine & Coastal resources, 2019).

Morphological identification based on organ remains, meat, bones, stained blood, and hair that had been collected from stranded marine mammals, is extremely difficult and may be impossible due to the high degree of degradation and the loss of many morphological features (Cousins et al., 2012). Access to an accurate and reliable tool for species identification of stranded carcasses is a critical task for forensic investigators (Ogden, Dawnay & McEwing, 2009). A wide variety of forensic tools specifically designed for non-specialists is now available for species identification. Tools for species identification can also be used for genetic studies and the study of wildlife analysis including in research involving whales and dolphins (Alacs et al., 2010; Baker et al., 2007; Baker et al., 2006; Linacre & Tobe, 2011; Lo et al., 2013; Lukoschek et al., 2009; Ogden, Dawnay & McEwing, 2009; Porter & Lai, 2017). Molecular markers, such as the random amplification of polymorphic DNA (RAPD), microsatellite, and mitochondrial DNA (mtDNA) genes, such as cytochrome *b* (CYTB) or cytochrome *c* oxidase I (COX-I), have been used to identify incidences of species trading of marine mammal species in the form of food products and stranded carcasses (Baker et al., 2007; Baker et al., 2006; Martinez & Daníelsdóttir, 2000; Sholl et al., 2013; Sholl et al., 2008; Silva et al., 2021; Teramitsu et al., 2000). The DNA sequences located between microsatellite regions, referred to as inter-simple sequence repeat (ISSR), are considered useful molecular markers that do not require prior DNA information (Steffler et al., 2016). ISSR is a dominant marker that can simultaneously and randomly generate multiple loci for a single primer which referred to as a DNA fingerprint. Additionally, ISSR markers have not yet been evaluated for marine mammal species discrimination.

Additionally, high resolution melting analysis (HRM) has been introduced as a rapid method for genotyping known variants and for the process of scanning for unknown variants (Wittwer, 2009). This approach has several notable advantages such as reducing the chances of cross-contamination and the fact that it does not require the handling of hazardous materials (Erdem et al., 2016; Gopaul et al., 2014). Additionally, it is recognized as being less time-consuming (Jiang et al., 2019). Besides, specimens could be distinguished by graph changes in the melting curves that are generated by HRM and are easily visualized (Jin et al., 2014). HRM can reveal results at different melting temperatures which can then be measured in real-time and reduce the subjective errors that are associated with human biases (Erali & Wittwer, 2010; Power, 1996; Reed, Kent & Wittwer, 2007; Tulsiani et al., 2010; Vossen et al., 2009; Wittwer et al., 2003). For example, ISSR markers coupled with HRM analysis or ISSR-HRM have recently been reported for the evaluation of several dog breeds (*Canis lupus familiaris*) in terms of their identification and classification (Kriangwanich et al., 2020). ISSR markers would be more effective in distinguishing between individuals and species due to the presence of a single primer that could produce multiple DNA bands from multiple loci (Gupta et al., 1994; Ng & Tan, 2015). Hence, the objective of this study was to evaluate the use of ISSR markers coupled with the HRM technique for the identification of marine mammal species. In addition, our ultimate objectives are to identify marine mammal species from stranded carcasses and to develop records and networks of stranded marine animals. The aim is to improve and correct the incidences of mismatches that are commonly associated with morphological identification. Moreover, researchers who utilize the marine mammal stranding network, expect to develop improved surveillance strategies and contribute to future prevention and conservation efforts.

## Materials and Methods

### Sample collection and DNA extraction

A total of 182 individual stranded marine mammal (cetaceans and dugong) carcasses were divided into 16 species (Table 1). The specimens used in this study were provided by veterinarians of the Phuket Marine Biological Center, Phuket, Thailand who had performed species identification based on external morphology. Skin samples were collected and preserved in 95% ethanol at −20 °C. The skin samples were obtained by using a sterile scalpel blade or scissors to yield a sampling size weighing approximately 25 mg and stored in sealed containers with 95% ethanol at −20 °C. Genomic DNA was extracted using a DNeasy Blood & Tissue Kit (QIAGEN GmbH, Hilden, Germany) following the manufacturer’s instructions. In brief, the skin samples were cut into small pieces and placed in 1.5 microcentrifuge tubes. Next, 180 μl of the ATL buffer and 20 μl of proteinase K were added. Samples were then mixed by vertexing and incubated at 56 °C until tissue samples were completely lysed. Finally, a 100 μl standard elution volume was added twice in order to recover the genomic DNA. The concentration, yield and purity values of the DNA were determined using a Beckman Coulter DU^®^ 730 spectrophotometer (Beckman Coulter, CA, USA). Lastly, DNA was kept at −20 °C for further analysis.

**Table 1:** Marine mammalian species accession number of Cytochrome *b* gene CYTB and number of samples for each of the 16 marine mammalian species.

Scientific name	Common name	Accession number of CYTB	Numbers
*Stenella longirostris*	Spinner dolphin	YP_009330816.1	31
*Stenella coeruleoalba*	Striped dolphin	YP_002586987.1	30
*Stenella attenuata*	Pantropical spotted dolphin	YP_002586893.1	28
*Tursiops aduncus*	Indo-Pacific bottlenose dolphin	YP_002587059.1	27
*Dugong dugon*	Dugong	NP_536770.1	24
*Pseudorca crassidens*	False killer whale	YP_007024877.1	7
*Steno bredanensis*	Rough-toothed dolphin	YP_009652599.1	7
*Lagenodelphis hosei*	Fraser’s dolphin	YP_009489122.1	7
*Grampus griseus*	Risso’s dolphin	YP_002587111.1	6
*Kogia sima*	Dwarf sperm whale	YP_009571934.1	6
*Neophocaena phocaenoides*	Indo-Pacific finless porpoise	YP_008082933.1	2
*Delphinus delphis tropicalis*	Long-beaked common dolphin	YP_002587098.1	2
*Sousa chinensis*	Indo-Pacific humpback dolphin	YP_002587046.1	1
*Kogia breviceps*	Pygmy sperm whale	NP_944671.1	1
*Ziphius cavirostris*	Cuvier’s beaked whale	YP_008081968.1	1
*Globicephala macrorhynchus*	Short-finned pilot whale	YP_007024890.1	1
Total			182

### Species confirmation and phylogenetic reconstruction

Species confirmation was performed by polymerase chain reaction (PCR) amplification using mtDNA D-loop long regions with (5′-CAT ATT ACA ACG GTC TTG TAA ACC-3′) as forward primer and (5′-GTC ATA AGT CCA TCG AGA TGT-3′) as reverse primer (Blair et al., 2014). PCR reactions were conducted in 25 µl reaction volumes using Platinum Taq DNA polymerase (Invitrogen, Waltham, MA, USA) and 10× reaction buffer. Generally, 2 mM MgCl_2_, 0.4 mg/ml bovine serum albumin, 0.25 mM dNTPs, 0.4 µM forward and reverse primers and 2 µl of the DNA template were used for PCR reactions. The PCR conditions were set as follows: 95 °C for 5 min, 40 cycles of 95 °C for 30 s for degeneration, 50 °C for 45 s for annealing and 72 °C for 1 min for extension. Finally, a final extension step employed conditions of 72 °C for 10 min. Sanger direct sequencing was then performed by Ward Medic Ltd., Bangkok, Thailand to obtain the DNA sequences. DNA were aligned and compared to those of the GenBank database to confirm species identification using external morphology.

The cytochrome b gene (CYTB) sequences were chosen because they displayed more accuracy in reconstructing animal phylogeny than the cytochrome c oxidase subunit 1 (COX-I). They are also known to exhibit relationships between species based on molecular and morphological analyses (Tobe, Kitchener & Linacre, 2010). Moreover, CYTB was chosen instead of the D-loop control region for phylogenetic reconstruction because the CYTB sequence displayed a better ability to resolve deep relationships when compared with the D-loop control region (Zhu et al., 1994). The phylogenetic relationships were reconstructed using Molecular Evolutionary Genetics Analysis or the MEGA X program (Kumar et al., 2018) with the use of the Maximum Likelihood (ML) and Tamura-Nei evolutionary model (Tamura & Nei, 1993) of the CYTB gene obtained from the NCBI GenBank database of all 16 marine mammals included in this study (Table 1). The initial tree used for the heuristic search was established automatically by applying Neighbor-joining and BioNJ algorithms to a matrix of pairwise distances estimated using the Tamura-Nei model. The topology was then established with the use of the superior log-likelihood value.

### Inter-simple sequence repeats coupled with high resolution melting analysis (ISSR-HRM)

DNA fingerprints were generated using ISSR markers because they are generally considered to be economical, easy-to-handle and can produce results quickly. This method has been confirmed in various studies involving genetic diversity, genetic structure and molecular identification (Sarwat, 2012). A total of 34 ISSR primers were screened to obtain a melting profile of the DNA fingerprints based on the ISSR fingerprints established by HRM referred to as “ISSR-HRM” (Kriangwanich et al., 2020). As a result, eight suitable ISSR primers that offered distinct melting curve patterns among all species were selected to create melting fingerprints using the ISSR-HRM technique (Table 2).

**Table 2:** Nucleotide sequences of Inter-simple sequence repeat (ISSR) primers obtained from the University of British Columbia.

Primers	Sequence (5′–3′)	Length
UBC812	GAG AGA GAG AGA GAG AA	17-mer
UBC817	CAC ACA CAC ACA CAC AA	17-mer
UBC818	CAC ACA CAC ACA CAC AG	17-mer
UBC826	ACA CAC ACA CAC ACA CC	17-mer
UBC827	ACA CAC ACA CAC ACA CG	17-mer
UBC847	CAC ACA CAC ACA CAC ARC	18-mer
UBC848	CAC ACA CAC ACA CAC ARG	18-mer
UBC880	GGA GAG GAG AGG AGA	15-mer

High resolution melting analysis coupled with ISSR markers (ISSR-HRM) was performed on PCRmax Eco 48 (PCRmax Limited, Staffordshire, UK) following the protocol established by Kriangwanich et al., 2020. This method was used with a final volume of 10 μl containing 1X using a SensiFast™ HRM kit (the EvaGreen^®^ dye, dNTPs and enhancers) (Bioline, Memphis, TN, USA), 0.5 μM ISSR primer and a 20 ng DNA template. Deionized water was added instead of the DNA template to establish a negative control. HRM analysis was carried out after 37 real-time PCR cycles at temperature increments of 0.1 °C/cycle between 55 °C and 95 °C to generate high resolution melting curves. Eco software v5.2.12 (PCRmax) was used to generate melt curve profiles from the ISSR-HRM, normalized curves, difference curves and amplification curves. All of this was performed to identify any existing differences between the melting profiles of 16 marine mammal species.

The temperature boundaries were set by the pre- and post-melt normalization regions after HRM analysis. This was done to generate the normalized melting curves (Wittwer et al., 2003). The melting temperature (T_m_) was primarily displayed by the melting curve for negative derivative of fluorescence (F) over temperature (T) with a normalized raw curve depicting a decreasing degree of fluorescence along with increasing temperatures. The melting profile of each marine mammal, referred to as a “melting fingerprint”, was achieved by implementing more than one melting peak from the ISSR-HRM technique. Hence, the theoretical interpretation of ISSR-HRM employed in this study would indicate that marine mammals in different species can contribute distinctive sets of multiloci leading to different sets of melting peaks based on a single primer.

### Accuracy and precision testing

Five marine mammals that had more than 20 samples (*n* > 20), including the Spinner dolphin (*Stenella longirostris*), Striped dolphin (*Stenella coeruleoalba*), Pantropical spotted dolphin (*Stenella attenuate*), Indo-Pacific bottlenose dolphin (*Tursiops aduncus*) and the Dugong (*Dugong dugon*), were selected to determine the accuracy and precision of our process by implementing a double-blind test. A total of 140 individuals from the five species mentioned above, each of which produced more than five samples per species, were used as an out-grouped template for species verification by ISSR-HRM. The known “melting fingerprint” of these five marine mammals established by the ISSR-HRM technique were applied with eight screened suitable ISSR markers to provide references of each species. Determination of the melting fingerprints was done based on T_m_ values of the melting peaks and the melting patterns of each individual when compared with the archived references. A true positive (TP), true negative (TN), false positive (FP) and false negative (FN) were used to calculate the percentage of accuracy ((TP+TN)/(TP+FP+FN+TN)), percentage of precision (TP/(TP+FP)), as well as sensitivity (TP/(TP+FN)) and specificity (TN/(TN+FP)) scores by using two-by-two confusion matrix from program R version 3.5.1. Melting fingerprints were independently established through a blind judging process administered by three qualified persons to achieve greater accuracy and precision.

## Results

### Phylogenetic reconstruction and ISSR-HRM melting profiles

**Figure 1:**
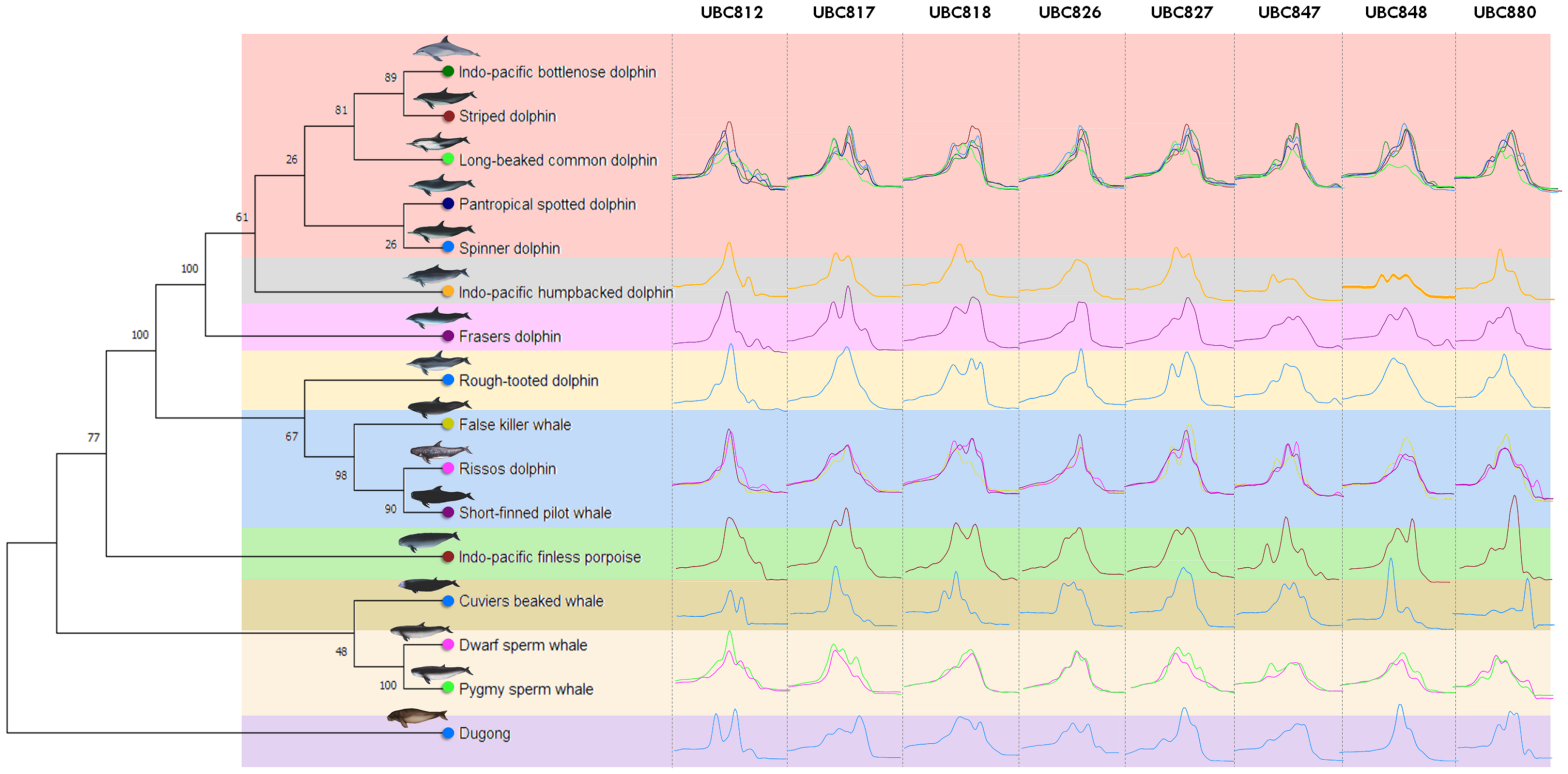
Genetic relationships among marine mammals included in this study presented by phylogenetic dendrogram based on data mining from NCBI GenBank of cytochrome *b* (CYTB) gene. Specifically, 16 marine mammal species were grouped according to their hierarchical five family taxonomic ranks; Dugonginae, Kogiidae, Ziphiidae, Phocoenidae and Delphinidae and divided into nine clusters (different color). The Family Delphinidae could be divided into another 5 clusters including the Indo-Pacific humpback dolphin (*Sousa chinesis*), Fraser’s dolphin (*Lagenodelphis hosei*) and the Rough-toothed dolphin (*Steno bredanensis*) which were all categorized separately, while the Indo-pacific bottlenose dolphin (*Tursiops aduncus*), Striped dolphin (*Stenella coeruleoalba*), Long-beaked common dolphin (*Delphinus delphis tropicalis*), Pantropical spotted dolphin (*Stenella attenuate*) and Spinner dolphin (*Stenella longirostris*) were grouped together and the False killer whale (*Pseudorca crassidens*), Risso’s dolphin (*Grampus griseus*) and the Short-finned pilot whale (*Globicephala macrorhynchus*) were all grouped in the same cluster. The ISSR-HRM melting curves of UBC812, UBC817, UBC818, UBC826, UBC827, UBC847, UBC848 and UBC880 are presented separately in each cluster (vertical direction).

The phylogenetic dendrogram was constructed of the CYTB gene obtained from the NCBI GenBank database of all 16 marine mammal species included in this study (Table 1, Supplemental File 3). The phylogenetic tree with the highest log-likelihood (−6081.46) is shown. The percentage of trees in which the associated taxa were clustered together is represented next to the branches (Fig. 1). There was a total of 1,140 positions in the final dataset. MtDNA D-loop sequences were used to firmly confirm all specimens of the stranded marine mammals to the correct species. All specimens (cetaceans and dugongs) revealed that they possessed a similar sequence to the specific species identified by the morphological characteristics of dead stranded cetaceans with an identity score of 96–100%.

A total of 34 ISSR primers were screened to generate DNA fingerprints and it was found that eight ISSR markers (23.53%) illustrated different ISSR fingerprints by PCR dispense multiple melting peaks or temperatures, as is shown in Table S1. The melting peak temperature among 16 marine mammal species obtained from eight different ISSR primers (UBC812, UBC817, UBC818, UBC826, UBC827, UBC847, UBC848 and UBC880) revealed 35, 29, 35, 37, 29, 44, 35 and 44 as the melting peaks established from each primer, respectively (Table 3). Additionally, all eight ISSR primers employing ISSR-HRM analysis were used to provide melting curves or “melting fingerprints” for all 16 marine mammal species at an annealing temperature of 58 °C. However, the melting fingerprints generated from UBC812, UBC817, UBC826 and UBC847 in the cluster comprised of the Indo-Pacific bottlenose dolphin, the Striped dolphin, the Long-beaked common dolphin, the Pantropical spotted dolphin and the Spinner dolphin were relatively similar; however, the melting peak temperatures differed among these marine mammals. This was likewise the case in the cluster comprised of the False killer whale, Risso’s dolphin and the Short-finned pilot whale (Fig. 1). For instance, the melting fingerprint of Indo-Pacific bottlenose dolphins generated from the UBC812 ISSR primer had four different melting peak temperatures at 80.0 °C, 81.9 °C, 86.1 °C and 88.0 °C. This outcome was determined to be reasonably related to the melting fingerprint of the Striped dolphin, which also revealed four distinctive melting peak temperatures of 79.9 °C, 82.1 °C, 86.1 °C and 88.0 °C, respectively. On the other hand, three other species of Delphinidae, namely the Indo-Pacific humpback dolphin, Fraser’s dolphin and the Rough-toothed dolphin, revealed unique melting fingerprints from UBC818, UBC827, UBC847, UBC848 and UBC880. Nonetheless, the melting fingerprints of 11 species of the Delphinidae Family in this study were achieved from most of the ISSR primers, especially for the T_m_ values, all of which were determined to be specifically varied when they were compared with each other.

**Table 3:** Percentage of accuracy, precision, sensitivity and specificity of each primer in four cetaceans and dugongs.

Primers		Spinner dolphin	Striped dolphin	Indo-Pacific bottlenose dolphin	Pan-tropical spotted dolphin	Dugong	Mean
UBC812	Accuracy	48.40	63.30	67.90	71.40	80.00	66.20
	Precision	52.00	65.00	63.00	61.00	79.00	64.00
	Sensitivity	76.47	76.47	85.71	91.67	100.00	86.06
	Specificity	14.29	46.15	50.00	56.25	16.67	36.67
UBC817	Accuracy	87.10	63.30	66.70	57.10	68.00	68.44
	Precision	89.00	61.00	69.00	56.00	71.00	69.20
	Sensitivity	96.15	87.50	94.74	93.33	94.44	93.23
	Specificity	40.00	35.71	0.00	15.38	0.00	18.22
UBC818	Accuracy	64.50	46.70	48.20	50.00	64.00	54.68
	Precision	67.00	42.00	48.00	46.00	63.00	53.20
	Sensitivity	95.24	83.33	92.31	91.67	100.00	92.51
	Specificity	0.00	22.22	7.14	18.75	10.00	11.62
UBC826	Accuracy	67.70	66.70	66.70	50.00	80.00	66.22
	Precision	70.00	68.00	71.00	48.00	79.00	67.20
	Sensitivity	95.45	89.47	89.47	100.00	100.00	94.88
	Specificity	0.00	27.27	12.5	6.67	16.67	12.62
UBC827	Accuracy	74.2	50.00	66.70	42.90	76.00	61.96
	Precision	73.00	46.00	67.00	42.30	75.00	60.66
	Sensitivity	100.00	84.62	94.12	91.67	100.00	94.08
	Specificity	11.11	23.53	20.00	6.25	14.29	15.04
UBC847	Accuracy	80.70	73.30	70.40	67.90	88.00	76.06
	Precision	81.50	73.00	66.70	63.60	91.30	75.22
	Sensitivity	95.65	88.89	93.33	93.33	95.45	93.33
	Specificity	37.50	50.00	41.67	38.46	33.33	40.19
UBC848	Accuracy	71.00	73.30	59.30	63.00	76.00	68.52
	Precision	74.10	78.90	60.00	48.00	78.30	67.86
	Sensitivity	90.91	78.95	93.75	100.00	94.74	91.67
	Specificity	22.22	63.64	9.09	16.67	16.67	25.66
UBC880	Accuracy	84.00	63.30	63.00	51.90	72.00	66.84
	Precision	84.60	62.50	60.80	52.20	70.80	66.18
	Sensitivity	95.65	88.24	93.33	85.71	100.00	92.59
	Specificity	50.00	30.77	25.00	15.38	12.50	26.73

**Figure 2:**
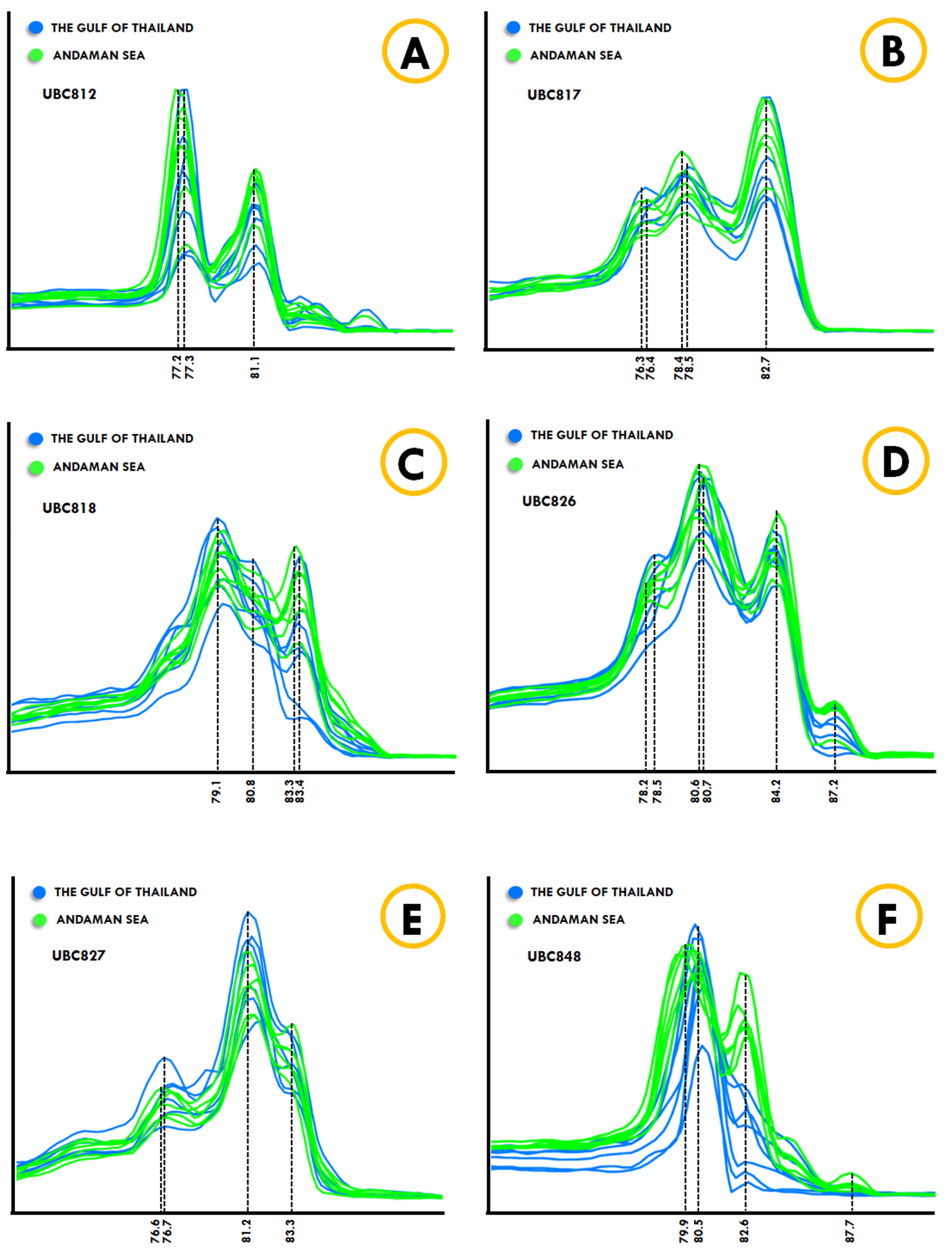
ISSR-HRM derivative melting curves of dugongs originating from the Andaman Sea (green) and the Gulf of Thailand (blue). A dotted line indicates melting temperatures. UBC812 (A), UBC817 (B), UBC818 (C), UBC826 (D), UBC827 (E) and UBC848 (F).

The *Kogia* spp., Dwarf sperm whale (*Kogia sima*) and Pygmy sperm whale (*Kogia breviceps*) generated moderately analogous melting fingerprints from all ISSR primers. Additionally, UBC826 created a very similar melting fingerprints for *Kogia* spp., although the findings were still diverse in terms of T_m_ temperatures at the second (80.6 °C and 80.3 °C) and third peaks (82.6 °C and 82.4 °C) of both the Dwarf sperm whale and the Pygmy sperm whale in the order already mentioned, while the Pygmy sperm whale also revealed an additional fourth melting peak temperature (87.2 °C). Results obtained from the phylogenetic tree based on the CYTB gene indicated that the Indo-Pacific finless porpoise and Cuvier’s beaked whale were each clustered into a single cluster (Fig. 1). Likewise, the melting curves obtained from the Indo-Pacific finless porpoise (*Neophocaena phocaenoides*) and Cuvier’s beaked whale (*Ziphius cavirostris*) were unique according to their different taxonomic family. Notably, the Dugong (*Dugong Dugon*) was separated into a single clade and had highly specific melting fingerprints generated from all ISSR markers that could clearly be used to distinguish them from all other cetaceans. Furthermore, two populations of dugong collected from the Gulf of Thailand (GOT) and the Andaman Sea (AND) were included in this study. According to our results, the melting fingerprints derived from UBC812, UBC817, UBC818, UBC826, UBC827 and UBC848 in dugongs obtained from different locations (GOT and AND) demonstrated that the T_m_ values or melting peak temperatures differed while the melting patterns were relatively comparable (Fig. 2). UBC812, UBC818 and UBC827 provided similar patterns of melting curves in comparisons made between the dugong populations, while the melting peak temperature was slightly different at 0.1 °C. However, three other ISSR primers provided different melting fingerprints of dugongs from AND and GOT. The first and second melting peaks of the melting fingerprints produced from UBC817 were different between the dugong populations originating from GOT and AND, as were those produced from UBC826. However, UBC848 generated a unique melting pattern for the population from AND with three different melting peaks recorded at 79.9 °C, 82.6 °C and 87.7 °C, while the population from GOT revealed only two melting peaks at 80.5 °C and 82.6 °C.

### Accuracy and precision testing

**Figure 3:**
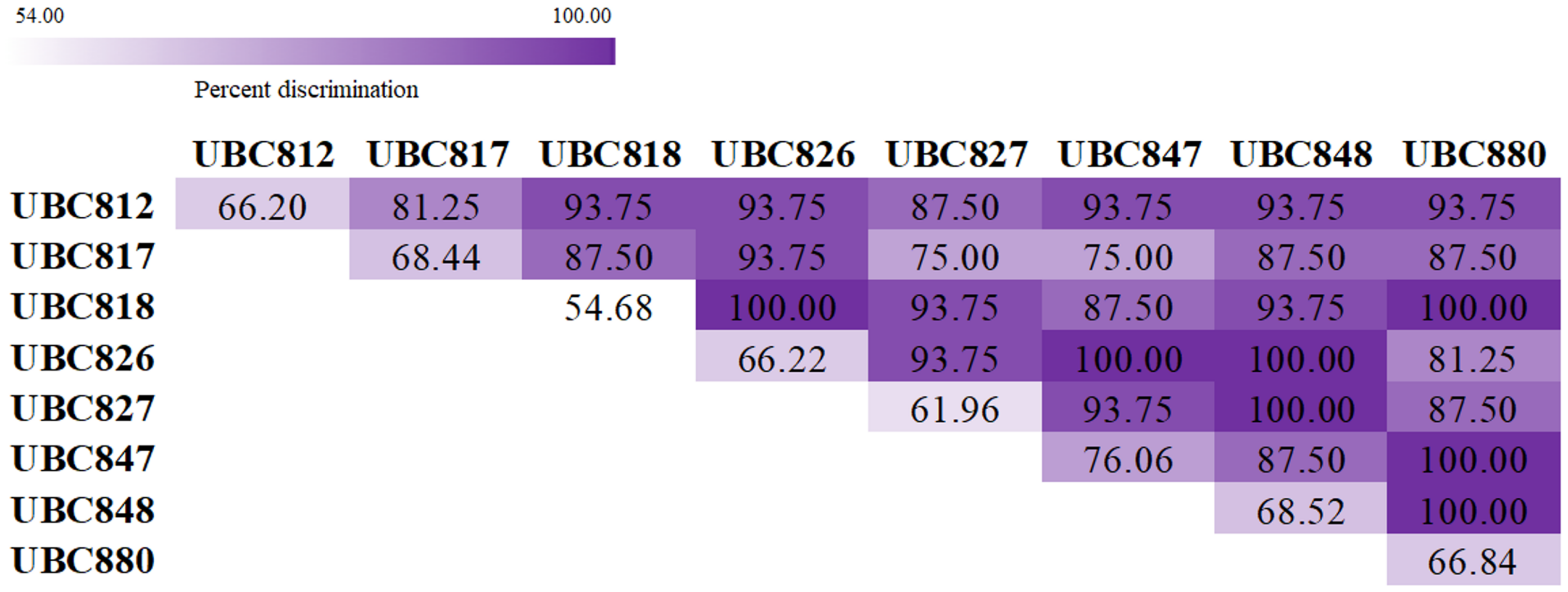
Heatmap showing percentage values for species discrimination from a combination of Inter-simple sequences repeat (ISSR) markers coupled with high resolution melting analysis (HRM) for the classification of marine mammals.

The melting fingerprints that were generated using the ISSR-HRM technique in this study could be used to develop and support an alternative method of species identification. The accuracy and precision testing of this technique were performed by three inspectors without recognition of any sample species information. The results presented in Table 3 suggest that UBC847 had the highest degree of mean accuracy and precision at 76.06% and 75.22%, respectively, with 93% sensitivity and 40% specificity values. Meanwhile, UBC818 was determined to be the least accurate with mean accuracy and precision values of 54.68% and 53.20%, respectively, and with 93% sensitivity and 12% specificity values, respectively. In addition, other ISSR primers revealed similar values in terms of the percentage of accuracy and the percentage of precision by approximately 60–70%. The Spinner dolphin, Striped dolphin and Indo-Pacific bottlenose dolphin reported percentage values of accuracy and precision within a range of 62–73%, with more than 90% sensitivity and more than 20% specificity (Table 4). Besides, the dugong was the most successfully identified species displaying values of 75.5% accuracy and 75.9% precision with values of 98% sensitivity and 15% specificity, whilst the Pantropical spotted dolphin was the most misclassified species displaying values of 56.78% for accuracy and 52.14% for precision with 93% sensitivity and 22% specificity. As is shown in Fig. 3, a total of 28 pairwise combinations of ISSR primers were used to generate the melting fingerprints that were used for species discrimination. Additionally, it was found that seven combinations could effectively identify 16 marine mammals with a 100% success rate when considering similar melting patterns obtained from a single primer.

**Table 4:** Mean percentage values of accuracy, precision, sensitivity and specificity of each of the five marine mammals.

Marine mammal species	Accuracy	Precision	Sensitivity	Specificity
Spinner dolphin	72.20	73.90	93.19	21.89
Striped dolphin	62.49	62.05	84.68	37.41
Indo-Pacific bottlenose dolphin	63.61	63.19	92.09	20.68
Pantropical spotted dolphin	56.78	52.14	93.42	21.73
Dugong	75.50	75.93	98.08	15.02

## Discussion

ISSR-HRM analysis conducted in this study resulted in the reliable identification of 16 marine mammal species using nuclear DNA extracted from skin samples. This was established to accurately identify and confirm the records of stranded marine mammals. Therefore, the highlights of this study include the contention that the melting fingerprints generated from ISSR-HRM analysis could be used as a straightforward and convenient alternative tool to facilitate the process of species identification for stranded marine mammals with a high degree of sensitivity. The outcomes of this study will offer benefits in terms of surveillance practices. They may also be used to update the existing stranding network and records, which could then be used in the development of appropriate future sustainable conservation strategies for marine mammals that inhabit Thai waters. The results could also be applied in investigations of illegal wildlife trading and help researchers to identify the factors that contribute to the causes of stranding.

Mitochondrial DNA (mtDNA) plays a major role in identification as the most commonly used marker as this marker exhibits many attributes that make it particularly well suited to marine mammal genetic diversity and differentiation studies (Martien et al., 2017; Rosel et al., 2017; Simon et al., 2006; Zink & Barrowclough, 2008). However, in 2017, Rosel et al., 2017 reported on some limitations associated with the use of mtDNA. An examination of the mtDNA gene sequence data obtained from pairs of closely-related cetacean populations, subspecies and species found that the determined subspecies-level was different from the actual subspecies pairs (Rosel et al., 2017). On the other hand, several studies demonstrated that the COX-I or DNA barcoding technique allowed for accurate identification of some species such as Blue whale (*Balaenoptera musculus*), Pygmy, Dwarf and Sperm whales (*Physeter macrocephalus*), common bottlenose dolphin (*Tursiops truncates*), Fraser’s dolphin, Risso’s dolphin, Striped dolphin, and Cuvier’s beaked whale (Alfonsi et al., 2013; Amaral, Sequeira & Coelho, 2007; Falcao et al., 2017; Silva et al., 2021; Viricel & Rosel, 2012). COX-I has been used in the identification of stranded rare species or deep-living species from highly degraded carcasses that could otherwise not be used to identify animals via their external morphology (Thompson et al., 2012). Also, COX-I can be applied to evaluate any intraspecific genetic variabilities and could be used to monitor population connectivity (Pauls et al., 2012). Additionally, nuclear DNA (microsatellite and SNPs), which are similar to the ISSR that we used in this study, can be effectively used in the assignment test and characterization of social structure, population differentiation and phylogenetic analysis of marine mammals (Bryant et al., 2012; Martien et al., 2017; Viricel et al., 2014). An advantage of using nuclear DNA (nDNA) is the diploid copy number, which makes it more predictive of nDNA profiling success and more relevant for identity testing and sample traceability than multi-copy and non-nuclear markers (Alonso et al., 2004; Walker et al., 2004). Hence, this nDNA approach provides multiple target sites within the DNA genome for the development of assays that can simultaneously and species-specifically generate DNA templates from a variety of species (Kanthaswamy et al., 2012; Ng et al., 2014).

The melting fingerprints generated by ISSR-HRM were used to discriminate between 16 cetaceans and dugongs and the melting patterns were significantly different based on a single primer obtained from each species. In addition, the family Delphinidae was previously determined to be the most morphologically and taxonomically diverse cetacean family, which consisted of six subfamilies (Rice, 1998). Accordingly, the genetic differences that were found to be significantly distinguishable could impact the melting fingerprints of cetaceans that were categorized in the same Delphinidae family. Morphologically and genetically, this family is known to be the most problematic due to the incomplete lineage sorting genes, such as COX-I, and possibly because of the introgression processes or insufficient time of divergence of some species within the family (Kessler, 2019; LeDuc, Perrin & Dizon, 1999; Perrin, Rosel & Cipriano, 2013; Silva et al., 2021; Zhou et al., 2011). On the other hand, the melting fingerprint of the Indo-Pacific bottlenose dolphin was comparable to dolphins in the genera *Stenella* and *Delphinus* based on the fact that the mitochondrial DNA of the Indo-Pacific bottlenose dolphin was more closely related to *Stenella* and *Delphinus* than to the common bottlenose dolphin (Charlton-Robb et al., 2011; LeDuc, Perrin & Dizon, 1999; Möller et al., 2008). However, the genetic study of the subfamily Delphininae has suggested that using anonymous nuclear markers and amplified fragment length polymorphism (AFLP) for phenetic analysis were found to be consistent with the morphological expectations required for identifying cetaceans (Kingston, Adams & Rosel, 2009). The results from the nuclear markers analysis were similar to our results, which found that the unique melting fingerprints were able to be used to distinguish between species members that belong to the subfamily Delphininae. Furthermore, the chromosome evolution indicated that the breakpoints that affected the function of genes related to kidney filtration, body development and immunity in the Indo-pacific humpback dolphin (Zhang et al., 2020) might be a reason for the individual melting patterns as well. The melting patterns in the same genera were more likely to be similar to each other, such as with the Dwarf sperm whale (*Kogia sima*) and the Pygmy sperm whale (*Kogia breviceps*). In addition, these two whales displayed very similar morphological characteristics except for the height and position of the dorsal fin (McAlpine, 2018). ISSR-HRM was used to effectively separate dugong populations that originated from the Andaman Sea and the Gulf of Thailand. Dugongs from different geographic regions are significantly differentiated from each other with the exception of those originating from East Africa, the Red Sea and the Arabian/Persian Gulf, which showed no significant differences to each other (Plon et al., 2019). Notably, skull morphology and morphometric measurement of dugongs originating from the Gulf of Thailand and the Andaman Sea were distinct from each other. Additionally, strong differentiations were observed between the dugong populations originating from the Gulf of Thailand and those originating from the North Andaman Sea when making a comparison of microsatellite alleles (Bushell, 2013; Nganvongpanit et al., 2017).

Nonetheless, the ISSR-HRM technique for species identification still has some limitations, especially with regard to the genetic variations associated with different populations of the same organism or animal. This could influence their genetic fingerprinting and may be used to identify differences in each population. This effect could also create discrepancies in the generation of melting fingerprints. For example, Pantropical spotted dolphins show high intraspecific morphological diversity, endemic subspecies and multiple populations in the eastern tropical Pacific Ocean (Courbis et al., 2014; Leslie, Archer & Morin, 2018); therefore, in this study, ISSR-HRM technique produced various melting fingerprints for the Pantropical spotted dolphins in each individual population. Consequently, we could have various melting fingerprints that could lead to lower percentage values of accuracy and precision. Moreover, the use of a single primer for species discrimination and classification could provide a degree of correction of around 70%. Thus, it was suggested that the pairwise combination could increase the accuracy and precision of two primers for species discrimination and classification.

Instead of using expensive analyses based on specific fluorescent probes or direct sequencing, immediate post-PCR analysis by HRM could provide a cheaper, selective and alternative method, especially when large sample sizes can be analyzed and when sequencing is outsourced (Ouso et al., 2020; Pena et al., 2012; Ramon-Laca et al., 2014; Reed, Kent & Wittwer, 2007; Vietina, Agrimonti & Marmiroli, 2013; Villinger et al., 2017; Wittwer, 2009). Sequencing is the preferred technique for any sequence analysis due to its high informational content (Naue, Hansmann & Schmidt, 2014). HRM is a fast and easy-to-handle technique (Wittwer, 2009) that is recognized as an alternative type of screening tool requiring a minimum number of processing steps. It is associated with reduced costs but can still provide the essential information needed for analysis. Additionally, the data interpretation process for the methods used in this study is both quick and reliable. Therefore, the examination of DNA remains fundamental for species investigation. The application of a successful method relies heavily on alternative methods that demand a minimum of procedures and costs, but which can still contribute to the important process of species identification (Cousins et al., 2012). Data interpretation of melting temperature or melting shape is the most important part of this technique as the analysis can be done visually by comparing the obtained normalized melting curves. The distinction between similar sequences obtained from the same animal species relies on differences in %GC content, amplicon length, type or position of base substitution, neighboring DNA of base substitution and the sequence itself, which could affect the melting behavior and lead to differences in melting fingerprints (Buddhachat, Kongket & Pandith, 2020; Garritano et al., 2009; Li et al., 2017; Schiwek et al., 2020; Tindall et al., 2009). The slight differences in melting temperature (0.1 °C) might have been caused by a variety of factors, such as the use of the pipetting technique, the DNA quality, the DNA template sequence being complementary to the primer, different batches being prepared in a single master mix solution and small changes in certain environmental factors (pH, ionic force, cation concentration, etc.) that arose from each PCR reaction (Slomka et al., 2017). Nevertheless, some primers, namely UBC812, UBC817, UBC826 and UBC847, cannot be used to create distinct melting fingerprints of marine mammals from the family Delphinidae. This determination probably resulted from variant compositions of the surrounding areas in the specific TATC or ATCT regions and would not be the result of specific changes in the HRM curves (Schmidt, Hulkkonen & Naue, 2016). Apparently, a better degree of discrimination for the melting temperature can be expected for A:T to G:C changes than for the neutral changes of A:T to T:A/G:C to C:G. This was due to the change from two to three hydrogen bonds which led to differences in T_m_ values between 0.8 °C and 1.4 °C in small amplicons (Liew et al., 2004). Notably, the limited number of samples may have led to certain limitations; for example, only one sample was available for some cetacean species. Nevertheless, these samples could still be used to identify differentiations among species.

In conclusion, the ISSR-HRM analysis developed in this study can be used as a stand-alone screening tool and serve as an effective, easy and rapid alternative tool for species discrimination and the classification of marine mammals at the species and subspecies level. However, reference controls should be recorded throughout the process to best facilitate data interpretation (Pelaez Sanchez et al., 2017). In this manner, the need to already have a species “in mind” and not only the appropriate animal group would need to be evaluated. The universal primers of ISSR can also provide a functionally initial overview and be used to potentially construct an alternative molecular tool for species identification (Hassan, Sabry & Ismail, 2020; Labastida et al., 2015). Altogether, the modular HRM analysis of nuclear gene DNA using ISSR primers from this study has proven to be a simple and reliable screening tool for a broad range of cetaceans and dugongs. Moreover, ISSR-HRM was proven to be a selective tool that could be practically used in laboratories and might be particularly useful in accurately identifying stranded marine mammals, especially certain cetaceans that possess very similar morphologies. However, an evaluation of this approach with higher numbers of samples per species might be required in further studies. Likewise, increasing numbers of marine mammal species should be used in order to develop a comprehensive database for species identification and discrimination. This would contribute to the development of future sustainable conservation strategies.

## Supplemental Information

10.7717/peerj.11689/supp-1 Supplemental Information 1 Melting temperatures from eight primers of ISSR-HRM analysis of each marine mammalian species.

10.7717/peerj.11689/supp-2 Supplemental Information 2 Melting temperatures of eight Inter-simple sequence repeat (ISSR) primers generated by High resolution melting analysis (HRM) of each individual marine mammal.

10.7717/peerj.11689/supp-3 Supplemental Information 3 The cytochrome b (CYTB) sequences that applied for phylogenetic reconstruction of 16 marine mammal species included in this study.

## Additional Information and Declarations

### Data Availability

The following information was supplied regarding data availability: The raw measurements are available in the Supplemental File.
