# Supplementary material for: Feasibility of melting fingerprint obtained from ISSR-HRM curves for marine mammal species identification
Source: PeerJ. 2021 Jun 25;9:e11689. doi: 10.7717/peerj.11689 (PMC8237827; doi:10.7717/peerj.11689)
Supplement: Supplemental Information 3 [file peerj-09-11689-s003.docx]

**The cytochrome b (CYTB) sequences that applied for phylogenetic reconstruction of 16 marine mammal species included in this study**

Spinner dolphin (*Stenella longirostris*)

>NC_032301.1:14185-15324 Stenella longirostris isolate z0000065 mitochondrion, complete genome

ATGACCAACATCCGAAAAACACACCCACTAATAAAAATCCTCAATGACGCATTCATTGATCTACCCACCC

CATCTAATATCTCCTCTTGATGAAATTTTGGTTCCTTACTAGGCCTTTGCCTAATTATACAAATCCTAAC

AGGTTTATTTCTAGCAATACATTACACGCCAGACACCTCAACTGCTTTCTCATCAGTCGCACATATCTGC

CGAGACGTCAACTATGGCTGATTTATCCGCTATCTACATGCAAACGGAGCCTCCATATTCTTCATCTGCC

TATACGCCCACATTGGACGTGGCCTGTACTATGGCTCCTATATATTCCAAGAAACATGAAACATTGGTGT

ACTCCTACTATTAACAGTCATAGCCACTGCATTCGTAGGCTACGTCCTNCCCTGAGGACAAATATCATTC

TGAGGCGCAACNGTCATCACCAANCTCCTATCAGCAATCCCTTATATTGGCACTACCNTAGTTGAATGAA

TCTGAGGTGGATTTTCCGTAGACAAAGCAACATTAACACGCTTTTTCGCTTTCCATTTTATCCTCCCATT

CATCATCACAGCATTAGCAGCCGTCCACCTACTATTCCTACACGAAACAGGATCCAACAACCCCACAGGA

ATCCCATCCAACATAGACATAATCCCATTCCACCCTTATTATACAATCAAAGACATCCTAGGTGGCTTAC

TCTTAATCTTAACCCTACTAGCACTAACCCTATTCACCCCTGACTTACTAGGAGACCCTGATAACTATAC

CCCAGCAAATCCACTAAGCACCCCTGCACACATCAAACCAGAATGATANTTCCTATTCGCATACGCAATC

TTACGATCAATCCCTAATAAACTTGGAGGAGTACTAGCATTATTACTCTCCATCCTTGTTCTAATCTTTA

TCCCAATACTTCAGACATCCAAACAACGAAGCATAATATTCCGACCCTTCAGCCAACTCCTATTTTGAAC

CNTAATCGCTGACCTCNTAACCTTAACATGAATTGGAGGCCAACCCGTAGAACACCCATATATCATTGTA

GGCCAATTAGCATCGATTCTATACTTCCTTCTAATCCTAGTGCTAATACCAACAGCCGGCCTCATTGAAA

ATAAACTCCTAAAATGAAGA

Striped dolphin (*Stenella coeruleoalba*)

>NC_012053.1:14195-15334 Stenella coeruleoalba mitochondrion, complete genome

ATGACCAACATCCGAAAAACACACCCACTAATAAAAATCCTCAATGACGCATTCATTGATCTACCCACTC

CATCTAATATCTCCTCTTGATGAAATTTTGGTTCCTTACTAGGCCTCTGCCTAATTATACAAATCCTAAC

AGGATTATTTCTAGCAATACATTACACGCCAGACACCTCAACTGCTTTCTCATCAGTCGCACACATCTGC

CGAGACGTCAACTATGGCTGATTCATCCGCTATTTACATGCAAACGGAGCCTCCATATTCTTCATCTGTC

TATACGCCCACATTGGACGTGGCCTATACTATGGCTCTTACATATTCCAAGAAACATGAAACATTGGTGT

ACTCTTACTACTAACAGTCATGGCCACTGCATTCGTAGGCTACGTCCTGCCCTGAGGACAAATATCATTC

TGAGGCGCAACCGTCATCACCAACCTCTTATCAGCAATCCCTTATATCGGCACTACCTTAGTCGAATGAA

TCTGAGGTGGATTCTCCGTAGACAAAGCAACATTAACACGCTTTTTCGCTTTCCACTTTATCCTCCCGTT

CATCATCACAGCATTAGCAGCCGTTCACCTGCTATTCTTACACGAAACAGGATCCAATAACCCAACAGGA

ATTCCATCCAACATAGACATAATCCCATTCCACCCTTATTATACAATTAAAGATATCCTAGGTGCCTTAC

TCCTAATCTTAACCCTACTAGCACTAACCCTATTCACCCCCGACCTACTAGGAGACCCTGATAACTATAC

CCCAGCAAATCCACTAAGCACCCCTGCACACATCAAACCAGAATGATACTTTCTATTCGCATACGCAATC

TTACGATCAATCCCTAACAAACTTGGAGGAGTCCTAGCACTACTACTTTCCATCCTTGTCCTAATCTTTA

TCCCAATACTCCAAACATCCAAACAACGAAGCATAATATTCCGACCCTTCAGCCAACTCCTATTTTGAAC

CCTAATCGCTGACCTCTTAACCTTAACATGAATTGGAGGCCAACCCGTAGAACACCCATATATCATTGTA

GGCCAATTAGCATCCATTTTATACTTCCTCCTAATCCTAGTGCTAATACCAACAGCCGGCCTTATTGAAA

ATAAACTCCTAAAATGAAGA

Pantropical spotted dolphin (*Stenella attenuate*)

>NC_012051.1:14195-15334 Stenella attenuata mitochondrion, complete genome

ATGACCAACATCCGAAAAACACACCCACTAATAAAAATCCTCAATGATGCATTCATTGATCTACCCACTC

CATCTAACATCTCCTCTTGATGAAATTTTGGTTCCTTACTAGGCCTCTGCCTAATTATACAAATCCTAAC

AGGATTATTTCTAGCAATACATTACACGCCAGACACCTCAACTGCTTTCTCATCAGTCGCACACATCTGC

CGAGACGTCAACTATGGCTGATTCATCCGCTATTTACATGCAAACGGAGCCTCCATATTCTTCATCTGTC

TATACGCCCACATTGGACGTGGCCTATACTATGGCTCTTATATATTCCAAGAAACATGAAACATTGGTGT

ACTCCTACTATTAACAGTCATAGCTACTGCATTCGTAGGCTACGTCCTGCCCTGAGGACAAATATCATTC

TGAGGCGCAACTGTCATCACCAATCTCCTATCAGCAATCCCTTATATTGGCACTACCTTAGTTGAATGAA

TCTGAGGTGGATTTTCCGTAGACAAAGCAACACTAACACGCTTCTTCGCTTTCCACTTTATCCTCCCGTT

CATCATCACAGCATTATCAGCCGTTCACTTGCTATTCCTACACGAAACAGGATCCAACAATCCCACAGGA

ATCCCATCCAACATAGACATAATCCCATTCCATCCTTATTATACAATCAAAGATATCCTTGGCGCTTTAC

TCTTAATCTTAACCCTACTAGCACTAACCCTATTCACCCCCGACCTACTAGGAGACCCTGACAACTATAC

TCCAGCAAATCCACTAAGCACCCCTGCACACATCAAACCAGAATGATACTTCCTATTCGCATACGCAATC

TTACGATCAATCCCTAATAAACTTGGAGGAGTACTAGCACTATTACTTTCCATCCTTGTCCTAATCTTTA

TCCCAATACTTCAAACATCCAAACAACGAAGCATAATATTCCGACCCTTCAGCCAACTCCTATTTTGAAC

CCTAATCGCTGACCTCTTAACCTTAACATGAATTGGAGGCCAACCCGTAGAACACCCATACATCATTGTA

GGCCAATTAGCATCCATCCTATACTTCCTCCTAATCCTAGTGCTAATACCAACAGCTGGCCTTATTGAAA

ATAAACTTCTAAAATGAAGA

Indo-Pacific bottlenose dolphin (*Tursiops aduncus*)

>NC_012058.1:14195-15334 Tursiops aduncus mitochondrion, complete genome

ATGACCAACATCCGAAAAACACACCCACTAATAAAAATCCTCAATGACGCATTCATTGATCTACCCACTC

CACCTAATATCTCCTCTTGATGAAATTTTGGTTCCTTACTAGGCCTTTGCCTAATTATACAAATCCTAAC

AGGATTATTTCTAGCAATACATTACACGCCAGACACCTCAACTGCTTTCTCATCAGTCGCACACATCTGC

CGAGACGTCAACTATGGCTGATTCATCCGCTATTTACATGCAAACGGAGCCTCCATGTTCTTCATCTGTC

TATACGCCCACATTGGACGTGGCCTATACTATGGCTCTTATATATTCCAAGAAACATGAAACATTGGTGT

ACTCTTACTACTAGCAGTCATAGCCACTGCATTTGTAGGCTACGTCCTGCCCTGAGGACAAATATCATTC

TGAGGCGCAACCGTCATCACCAACCTCTTATCAGCAATCCCTTATATTGGCACTACCTTAGTCGAATGAA

TCTGAGGTGGATTCTCCGTAGACAAAGCAACACTAACACGCTTTTTCGCTTTCCACTTTATCCTCCCGTT

CATCATCACAGCATTAGCGGCCGTTCACCTGCTATTCCTACACGAAACAGGATCCAATAACCCCACAGGA

ATCCCATCCAATATAGACATAATCCCATTCCACCCTTATTATACAATCAAAGACATCCTAGGTGCCTTAC

TCCTAATCTTAACCCTACTAGCACTAACCCTATTCACCCCCGACCTACTAGGAGACCCTGATAACTATAC

CCCAGCAAATCCACTAAGCACCCCTGCACACATCAAACCAGAGTGATACTTTCTATTCGCATACGCAATC

TTACGATCAATCCCTAATAAACTTGGAGGAGTCCTAGCACTACTACTTTCCATCCTCGTCCTAATCTTTA

TCCCAATACTTCAAACATCCAAACAACGAAGCATAATATTCCGACCCTTCAGCCAACTCCTATTTTGAAC

CCTAATCGCGGACCTCTTAACCTTAACATGAATTGGAGGCCAACCCGTAGAACACCCATATATCATTGTA

GGCCAATTAGCATCTATCCTATACTTCCTCCTAATCCTAGTGCTAATACCAACAGCCGGCCTTATTGAAA

ATAAACTCCTAAAATGAAGA

Dugong (*Dugong dugon*)

>NC_003314.1:14155-15294 Dugong dugon mitochondrion, complete genome

ATGACCAACATCCGAAAATCACACCCACTAATCAAAATCCTAAACAACTCCTTCATTGACCTCCCTACCC

CCGTAAATATCTCATCATGATGAAACTTTGGCTCCCTACTCGGGGCATGCCTGATTATTCAAATTCTCAC

AGGATTATTCCTGGCCATACACTACACATCAGACACACTAACCGCATTCTCCTCAGTAACCCATATTTGC

CGGGATGTAAACTACGGCTGAATTATTCGATATCTTCACGCTAACGGGGCATCAATATTCTTCCTATGCC

TCTACGCCCACATTGGACGCGGAATCTATTACGGCTCATACCTATATCCAGAAACCTGAAACATTGGTAT

CGTACTGCTACTCACAGTTATAGCTACTGCCTTCATAGGGTACGTCCTCCCATGAGGACAAATATCATTC

TGAGGAGCAACCGTTATTACTAACCTCCTGTCAGCTATCCCCTACATCGGCACCAACCTAGTCGAATGAG

TTTGAGGGGGATTCTCAGTAGACAAAGCCACCCTCACCCGATTCTTCGCCCTACACTTCATCCTACCCTT

CATCGTAACCGCCCTAGTAATAGTCCACTTACTATTCCTCCACGAAACAGGCTCCAACAACCCCACGGGA

CTGATCTCCGACTCAGACAAAATCCCATTCCACCCATATTATTCAGTCAAAGACCTCCTAGGCCTATTCC

TCCTCATTCTAGTCTTACTCCTACTAACCCTGTTCTCCCCGGACATACTGGGAGACCCAGACAACTACAC

ACCAGCCAACCCACTAAACACCCCTCCCCACATTAAACCAGAATGATACTTTCTATTCCGATACGCTATC

CTCCGATCTATCCCTAATAAACTAGGCGGCGTGTTAGCCCTCGTACTCTCCATCCTAATCCTAGCGCTCC

TCCCACTCCTCCACACATCCAAACAACGAAGCCTATCATTCCGACCTCTAAGCCAATGCCTCTTTTGAAT

TCTGGTAGCCGACCTGATCACACTCACATGAATCGGCGGCCAACCAGTAGAACATCCCTACATCATCATC

GGCCAACTAGCCTCAATCCTGTACTTCTCCATCATCCTCATCTTTATGCCCATCGCAGGCCTAATTGAAA

ATCACCTACTTAAATGAAGG

False killer whale (*Pseudorca crassidens*)

>NC_019577.1:14201-15340 Pseudorca crassidens mitochondrion, complete genome

ATGACCAACATCCGAAAAACACACCCACTAATAAAAATTATCAATAACGCATTCATTGACCTACCCACTC

CATCTAACATCTCCTCATGATGAAACTTTGGCTCCCTACTAGGCCTCTGCCTAATCATACAAATCCTAAC

AGGTTTATTCCTAGCAATACACTACACGCCAGACACCTCAACCGCTTTTTCATCAGTCGCACACATCTGT

CGAGACGTCAACTATGGCTGATTCATCCGCTACCTACATGCAAACGGAGCCTCCATATTCTTCATCTGCC

TTTATGCCCACATCGGACGTGGCTTATACTATGGCTCTTATATATTCCAAGAAACATGAAACATTGGCGT

GCTTCTGCTACTAGCAGTCATAGCCACTGCATTCGTAGGCTATGTTCTACCCTGAGGACAGATATCATTC

TGAGGCGCAACCGTCATCACCAATCTTCTATCAGCAATCCCCTACATCGGTACCACTTTAGTAGAATGAA

TCTGAGGAGGATTTTCCGTAGACAAAGCAACACTAACACGTTTTTTCACTCTCCACTTTATCCTCCCATT

CATCATTACAGCACTAACAGCTACCCACCTACTATTCCTACACGAGACTGGATCCAATAACCCCACAGGA

ATCCCATCCAACATAGACATAATTCCATTCCACCCTTATTACACAATTAAAGATATCCTAGGCGCCCTAC

TCTTAATTCTAACACTACTAACACTAACCCTATTCACCCCCGACCTACTAGGAGACCCTGATAACTATAT

TCCAGCAAACCCACTAAACACCCCTGCACACATCAAACCAGAATGATATTTCCTATTCGCGTATGCAATC

TTACGATCAATTCCTAATAAACTTGGAGGAGTACTAGCATTACTACTTTCCATTCTTATCCTAATCTTTA

TCCCAATACTTCAAACATCCAAACAACGAAGCATAATATTCCGCCCCTTTAGCCAACTTCTATTTTGAAC

TCTAATCGCAGACCTCCTAACCTTAACATGAATTGGAGGCCAACCTGTAGAACACCCATACATCATCCTA

GGCCAATTAGCATCTATCTTATACTTCCTCCTAATCTTAGTGCTAATACCAACAGTCAGCCTTATTGAAA

ATAAACTTCTAAAATGAAGA

Rough-toothed dolphin (*Steno bredanensis*)

>NC_042761.1:14194-15333 Steno bredanensis voucher CRI008296 mitochondrion, complete genome

ATGACCAACATCCGAAAAACACACCCACTAATAAAAATCCTCAATGACGCATTCATTGACCTGCCCACTC

CATCTAACATTTCTTCATGATGAAACTTTGGCTCCTTGCTAGGCCTCTGCCTAATTATACAAATCCTAAC

TGGTTTATTTCTAGCAATACACTACACACCGGACACCTCTACCGCTTTTTCATCAGTCGCACACATCTGT

CGAGACGTCAACTATGGCTGATTCATCCGCTATTTACATGCAAACGGAGCTTCCATATTCTTCATCTGCC

TTTACGCCCATATTGGACGTGGCCTCTATTATGGCTCTTATATATTCCAAGAAACATGAAACATCGGCGT

ACTCCTACTACTAACAGTCATAGCCACTGCATTTGTAGGCTACGTTTTACCCTGAGGACAAATATCATTC

TGAGGTGCAACCGTCATTACCAACCTCCTATCAGCAATCCCTTACATCGGCACTACCTTGGTAGAATGAA

TCTGAGGCGGATTTTCCGTAGACAAAGCAACACTAACACGTTTTTTCGCTTTCCACTTTATCCTCCCATT

CATCATCATAGCATTAGCAACTGTCCACCTACTATTCCTACACGAGACAGGATCCAACAATCCCACAGGA

ATCCCATCCAACATAGATATAATCCCATTCCACCCTTATTACACAATCAAAGACATCCTAGGCGCCTTAC

TTTTAATCCTAACTTTACTAGCACTAACCCTATTCACCCCCGACCTACTAGGAGACCCCGACAACTATAC

CCCAGCAAATCCACTAAGCACCCCTGCACACATCAAACCAGAATGGTATTTCCTATTCGCATACGCAATC

TTACGATCAATCCCCAACAAACTTGGAGGAGTACTAGCACTACTACTTTCCATCCTTATCCTAATCTTTA

TCCCAATACTTCAAACATCCAAACAACGAAGCATAATATTCCGACCCTTTAGCCAACTCTTATTTTGAAC

CCTAATCGCAGACCTCCTAACCTTAACATGAATTGGGGGCCAACCTGTAGAACACCCATACATCATTGTA

GGCCAATTAGCATCTATTTTATATTTCCTCCTAATCCTAGTGCTAATACCAGCAGTCGGCCTTATTGAAA

ATAAACTCCTAAAATGAAGA

Fraser’s dolphin (*Lagenodelphis hosei*)

>NC_037848.1:14195-15334 Lagenodelphis hosei voucher CRI000009 mitochondrion, complete genome

ATGACCAACATTCGAAAAACACACCCACTAATAAAAATCCTCAATGACGCATTCATTGATCTACCCACTC

CATCTAATATCTCCTCTTGATGAAATTTTGGTTCCTTGCTAGGCCTCTGCCTAATTATACAAATCCTAAC

AGGATTATTTCTAGCAATACATTACACGCCAGACACCTCAACCGCTTTCTCATCAGTCGCACACATCTGC

CGAGACGTCAACTATGGCTGATTCATCCGCTATTTACATGCAAACGGAGCCTCCATATTCTTCATCTGCC

TATACGCCCATATTGGACGCGGTTTATACTATGGCTCTTATATATTCCAAGAAACATGAAACATTGGTGT

ACTCCTACTATTAACAGTCATAGCCACTGCATTCGTAGGATACGTCCTGCCCTGAGGACAAATATCATTC

TGAGGCGCAACCGTCATCACCAACCTTCTATCAGCAATCCCTTATATCGGTACTACCTTAGTTGAATGAA

TCTGAGGTGGATTTTCCGTAGATAAAGCAACATTAACACGCTTTTTCGCTTTCCACTTTATCCTCCCATT

CATCATCACAGCATTAGCAGCCGTTCACCTATTATTCCTACACGAAACAGGATCCAACAATCCCACAGGA

ATCCCATCCAACATAGACATAATCCCATTCCACCCTTATTACACAATCAAAGACATCCTAGGTGCCTTGC

TCCTAATCCTAACCTTACTAGCACTAACCCTATTCACCCCCGACCTACTAGGAGACCCTGACAATTATAC

TCCAGCAAACCCACTAAGCACCCCTGCACACATTAAACCAGAATGATACTTCCTATTTGCATACGCAATC

TTACGATCAATCCCTAATAAACTCGGAGGAGTACTAGCACTATTACTCTCCATCCTCGTTCTAATCTTTA

TCCCAATACTTCAGACATCCAAACAACGAAGCATAATATTCCGACCCTTCAGCCAACTCCTATTTTGAAC

CCTAATCGCTGATCTCCTAACCTTAACATGAATTGGAGGCCAACCCGTAGAACACCCATACATCATTGTA

GGCCAATTAGCATCTATTCTATACTTCCTCCTAATCCTAGTGCTAATACCAACAGCTGGCCTTATTGAAA

ATAAACTCCTAAAATGAAGA

Risso’s dolphin (*Grampus griseus*)

>NC_012062.1:14197-15336 Grampus griseus mitochondrion, complete genome

ATGACCAACATCCGAAAAACACACCCACTAATAAAAATCATCAATAACGCATTCATTGATCTACCCACTC

CATCTAACATTTCCTCATGATGAAACTTTGGCTCCTTACTAGGCCTCTGCCTAATCATACAAATCCTAAC

AGGTCTATTCCTAGCAATACATTACACGCCAGACACCTCAACTGCTTTCTCATCAGTCGCACACATCTGT

CGAGACGTCAACTATGGCTGATTCATCCGCTACCTACATGCAAACGGAGCCTCCATATTCTTCATCTGCC

TTTACGCCCACATCGGACGTGGCTTATATTATGGCTCTTATATATTTCAAGAAACATGAAACATTGGTGT

ACTCCTATTACTAACAGTTATAGCTACTGCATTCGTAGGCTATGTTCTACCCTGAGGACAAATATCATTC

TGAGGCGCAACCGTCATCACCAATCTCCTATCAGCAATCCCCTACATCGGTACTACTTTAGTAGAATGAA

TCTGAGGTGGATTTTCCGTAGACAAAGCAACACTAACACGCTTTTTCGCTTTCCACTTTATCCTCCCATT

CATCATCACAGCATTAGTAGCTGTTCACCTGCTATTCCTACACGAGACAGGATCCAATAACCCCACAGGA

ATCCCATCCAACATAGACATAATTCCATTCCACCCCTATTACACAATTAAAGACATCCTGGGCGCCCTAC

TCCTAATCCTAACACTACTAACACTAACCCTATTCACCCCTGACCTACTAGGAGACCCTGATAACTACAC

TCCAGCAAACCCGCTAAGCACCCCTGCACACATCAAACCAGAATGATATTTCCTATTCGCATATGCAATC

TTGCGATCAATTCCCAACAAACTTGGAGGAGTACTAGCACTATTACTTTCCATCCTTATCCTAATCTTTA

TCCCAATACTTCAAACATCCAAACAACGAAGCATAATATTCCGTCCCTTTAGCCAACTCTTATTCTGAAC

CCTAATCGCAGACCTCCTAACCTTAACATGAATTGGAGGCCAACCCGTAGAACATCCGTACATCATTGTA

GGTCAATTAGCATCTATCTTATATTTCCTCCTAATCTTAGTGCTAATACCAACAATCAGCCTTATTGAAA

ATAAACTCCTAAAATGAAGA

Dwarf sperm whale (*Kogia sima*)

>NC_041303.1:14187-15326 Kogia sima mitochondrion, complete genome

ATGACCAACATCCGAAAAACACACCCACTGATAAAAATCATCAACAACGCATTTGTCGACCTCCCCACTC

CATCAAACATCTCCTCATGATGAAACTTCGGCTCCCTACTCGGCCTATGTCTCATTACACAGATCCTAAC

AGGCCTGTTTCTAGCGATACACTACACCCCAGATACAACAACCGCTTTCTCATCAGTCACACACATTTGC

CGAGATGTCAACTATGGCTGGGTAATCCGATATTTACACGCAAATGGAGCCTCCATGTTCTTCATCTGCC

TCTACGCCCACGTAGGACGTGGCCTATACTACGGCTCCTATGTTTTCCAAGAAACATGAAACATCGGAGT

AATCTTACTATTTACAGTAATAGCCACCGCATTCGTAGGCTACGTCCTGCCCTGAGGCCAAATATCATTC

TGAGGAGCAACCGTCATCACAAACCTTATATCCGCAATCCCTTACATCGGCACCACCCTAGTGGAATGAG

TTTGAGGTGGCTTCTCCGTGGACAAAGCTACGCTAACACGCTTCTTTGCTTTCCACTTTATTCTCCCCTT

CATCATCCTAGCACTAGCAATAGTCCACCTCCTATTTCTCCACAAAACAGGATCCAACAACCCCCTAGGA

ATTCCTTCTGATATAGACAAAATCCCATTCCACCCCTACTACACAATCAAAGATATCCTAGGCGCCCTAC

TACTAATCTCAGCACTACTCACACTGACCCTGTTCGCACCTGATCTACTAGGAGACCCCGACAACTATAC

CCCAGCAAACCCACTAAGCACCCCCGCACACATTAAACCAGAATGATATTTTCTGTTCGCATACGCCATT

CTACGATCAATTCCCAACAAACTGGGAGGCGTCATAGCCCTACTCCTCTCCATCCTGGTCCTAATACTCA

TCCCAATACTCCACACCTCCAAACAACGAAGCATAATATTCCGGCCCTTCAGCCAATTCTTATTCTGAGT

CTTAGTTGCAGACCTACTGACCTTAACATGAATTGGAGGCCAACCCGTAGAACACCCATACATAACCCTA

GGTCAACTAGCATCCACCCTATATTTTCTCCTGATTCTAGTCCTAATACCACTAACTAGCCTCATCGAGA

ATAAACTCTTAAAATGAAGA

Indo-Pacific finless porpoise (*Neophocaena phocaenoides*)

>NC_021461.1:14196-15335 Neophocaena phocaenoides mitochondrion, complete genome

ATGACCAACATCCGGAAAACACACCCACTAATAAAAATTATTAACAACGCACTTATCGATCTCCCCGCCC

CATCAAACATCTCCTCGTGATGAAATTTTGGCTCCTTACTAGGCCTCTGCCTGATCATACAAATCCTAAC

AGGCCTATTTCTAGCAATACACTACACACCAGACACCTCTACCGCCTTTTCATCAGTCACACACATCTGT

CGAGACGTAAATTACGGCTGAATCATTCGATACCTCCACGCAAACGGAGCCTCTATGTTCTTCATTTGCC

TCTATATCCACATCGGACGTGGCCTGTATTACGGCTCCTACATATTCCAAGAAACATGAAATATTGGTGT

ACTCCTACTACTAATAGTCATAGCCACCGCATTTGTAGGCTACGTTCTACCCTGAGGGCAAATATCATTT

TGAGGTGCTACCGTCATCACAAACCTCTTGTCAGCAATCCCTTATATTGGCAGCACACTAGTGGAGTGGA

TCTGAGGCGGGTTCTCTGTAGACAAAGCAACATTAACACGTTTCTTCGCCTTCCACTTTATTCTCCCATT

CATCATTACAGCACTAATAATCGTCCACTTACTATTTCTCCACGAAACAGGCTCAAACAACCCCACAGGA

ATTCCGTCCAACATAGACATAATCCCCTTTCACCCTTATTATACAATCAAGGACATCCTAGGTATCCTAC

TATTAATCCTGACCTTACTAACACTAACCTTATTTTCACCCGACCTTCTAGGTGACCCTGATAACTATGT

CCCAGCAAACCCACTGAGCACTCCAGCACACATTAAACCAGAGTGATATTTCCTCTTCGCATATGCAATC

CTACGATCAATCCCCAACAAACTGGGGGGAGTACTAGCATTACTACTTTCCATCCTCATCCTAGTACTAG

TCCCAATACTCCAAACCTCCAAACAACGAAGCATGATATTTCGGCCCTTTAGCCAACTCCTATTTTGAAC

TTTAATTGCAGACCTCTTAACCCTAACATGAATCGGAGGTCAGCCTGTAGAACACCCGTATATTATCGTA

GGCCAATTAGCATCTATCTTATATTTCCTTTTAATTCTAGTGCTAATACCAACAGCCAGCATTATTGAGA

ACAAACTCCTAAAATGAAGA

Long-beaked common dolphin (*Delphinus delphis tropicalis*)

>NC_012061.1:14194-15333 Delphinus capensis mitochondrion, complete genome

ATGACCAACATCCGAAAAACACACCCGCTAATAAAAATCCTCAATGATGCATTCATTGATCTACCCACTC

CATCTAATATCTCCTCTTGGTGAAATTTTGGTTCCTTACTAGGCCTCTGCCTAATTATACAAATCCTAAC

AGGACTATTTCTAGCAATACATTACACGCCAGATACCTCAACTGCTTTCTCATCAGTCGCACACATCTGC

CGAGACGTCAACTATGGCTGATTCATCCGCTATTTACATGCAAACGGAGCCTCCATATTCTTCATCTGTC

TATACGCCCACATTGGACGTGGCCTATACTATGGCTCTTATATGTTCCAAGAAACATGAAATATTGGTGT

ACTCTTACTACTAACAGTCATAGCCACTGCATTCGTAGGTTACGTCCTACCCTGAGGACAAATATCATTC

TGAGGCGCAACCGTCATCACCAACCTCTTATCAGCAATCCCTTATATTGGCACTACCTTAGTTGAATGAA

TCTGAGGTGGATTCTCCGTAGACAAAGCAACATTAACACGCTTTTTCGCTTTCCACTTTATCCTCCCATT

CATCATTACAGCATTAGCAGCCGTTCACCTGCTATTCCTACACGAAACAGGATCCAATAACCCCACAGGA

ATCCCATCCAATATAGACATAATCCCATTCCACCCTTATTACACAATCAAAGATATCCTAGGTGCCTTAC

TCCTAATCTTAACCCTACTAGCACTGACCCTATTCACTCCAGACCTACTAGGAGACCCTGATAACTATAC

CCCAGCAAATCCACTAAGCACCCCTGCACATATCAAACCAGAATGATACTTTCTATTCGCATACGCAATC

TTACGATCAATCCCTAATAAACTTGGAGGAGTCCTAGCACTACTACTCTCCATCCTTATCCTAATCTTTA

TCCCAATACTTCAAACATCCAAACAACGAAGCATAATATTCCGACCCTTCAGCCAACTCCTATTTTGAAC

CCTAATCGCTGACCTCTTAACCTTAACATGAATTGGAGGTCAACCCGTAGAACACCCATATATCATTGTA

GGCCAATTAGCATCTATTCTATACTTCCTCTTAATCCTAGTGCTAATACCAACAGCCGGCCTTATTGAAA

ATAAACTCCTAAAATGAAGA

Indo-Pacific humpback dolphin (*Sousa chinensis*)

>NC_012057.1:14197-15336 Sousa chinensis mitochondrion, complete genome

ATGACCAACATCCGAAAAACACACCCACTAATAAAAATCCTCAATGACGCATTCATTGATCTACCCACTC

CATCTAATATCTCCTCTTGATGAAATTTTGGTTCCTTACTAGGTCTCTGCCTAATCATACAAATCCTAAC

AGGATTATTCCTAGCAATACATTACACGCCAGACACCTCAACTGCTTTCTCATCAGTCGCACACATCTGC

CGAGACGTCAACTATGGCTGATTCATCCGCTATTTACATGCAAACGGAGCCTCCATATTCTTCATCTGTC

TATACGCCCACATTGGACGTGGCCTATACTATGGCTCTTATATGTTCCAAGAAACATGAAACATTGGTGT

ACTCCTGCTATTAACAGTCATAGCCACTGCATTCGTAGGCTACGTCCTGCCCTGAGGACAAATATCATTC

TGAGGCGCAACCGTCATCACCAACCTCCTATCAGCAATCCCTTATATTGGCACTACCTTAGTTGAATGAA

TCTGAGGTGGATTTTCCGTAGACAAAGCAACATTAACACGCTTTTTCGCTTTCCACTTTATCCTTCCCTT

CATCATCACAGCATTAGCAGCCGTTCACCTGCTATTCCTACATGAAACAGGATCCAACAACCCTACAGGA

ATTCCATCCAACATAGACATAATCCCATTTCACCCCTATTATACAATCAAAGACATCCTAGGTGCCTTAC

TCTTAATCTTAACCCTACTAGCACTAACCCTATTCACCCCCGACCTACTAGGAGACCCTGATAACTACAC

CCCAGCAAATCCACTAAGCACCCCTGCACACATCAAACCAGAATGAGATTTCCTATTCGCATACGCAATC

TTACGGTCAATCCCTGATAAACTTGGAGGAGTACTAGCACTACTACTTTCCATCCTTATCCTAATCTTTA

TTCCAATACTTCAGACATCCAAACAACGAAGCATAATATTCCGTCCCCTCAGCCAACTCCTATTTTGAAC

CCTAATCGCTGACCTCTTAACCTTAACATGAATTGGAGGCCAACCCGTAGAACATCCATATATCATTGTA

GGTCAATTAGCATCTATTTTATACTTCCTCCTAATCCTAGTGCTAATACCAACAGCCGGCCTTATTGAAA

ATAAACTCCTAAAATGAAGA

Pygmy sperm whale (*Kogia breviceps*)

>NC_005272.1:14191-15330 Kogia breviceps mitochondrion, complete genome

ATGACCAACATCCGAAAAACACACCCATTGATAAAAATCGTCAACAACGCATTCATCGACCTCCCCACTC

CATCAAACATCTCCTCATGATGAAATTTCGGCTCCCTGCTTGGCCTGTGTCTCATCACACAAATCCTAAC

AGGCCTATTTCTAGCAATACACTATACACCAGACACAACAACCGCCTTCTCATCAATCACACATATTTGT

CGAGACGTTAATTACGGCTGAGTAATCCGATACCTACACGCAAACGGAGCCTCCATATTCTTCATCTGCC

TCTACGCCCACGTAGGGCGGGGCCTATATTATGGCTCCTACACTTTCCAAGAAACATGAAACATCGGAGT

GGTTCTGCTATTTACAGTAATGGCCACCGCATTTGTAGGCTACGTCCTACCCTGAGGCCAAATATCATTC

TGAGGAGCAACCGTCATCACCAACCTTATATCCGCAATTCCTTATATCGGCACCACCCTAGTAGAATGAG

TCTGAGGTGGCTTCTCCGTAGACAAAGCCACATTAACACGCTTCTTTGCCTTTCACTTCATCCTCCCCTT

TATCATCCTAGCACTGGCAATGGTCCACCTCTTATTCCTCCACGAAACAGGATCCAACAACCCCATAGGA

ATCCCATCCGACATAGACAAAATCCCATTCCACCCCTACTACACAATCAAGGACATCTTAGGCGCCCTAC

TGCTAATCTCAGCACTACTCACATTAACCCTATTCGCACCAGACCTATTAGGAGACCCTGACAACTACAC

CCCAGCAAACCCACTAAGCACCCCGGCACACATTAAACCAGAATGATATTTCCTATTTGCATATGCCATC

CTACGATCCATCCCTAACAAACTAGGGGGAGTCCTAGCCCTACTCCTCTCCATTCTAATCCTGATACTCA

TCCCAATACTCCACACCTCCAAACAACGAAGCATAATATTCCGACCCTTCAGCCAATTCTTATTCTGAAC

CCTAGTTGCAGACCTACTAACCTTGACATGAATTGGGGGCCAACCCGTGGAACACCCATACGTAACCCTA

GGCCAACTAGCATCCATCCTATACTTCCTCCTAATTCTAGTCCTAATGCCACTGACTAGCCTCATTGAGA

ACAAACTCTTAAAATGAAGA

Cuvier’s beaked whale (*Ziphius cavirostris*)

>NC_021435.1:14195-15334 Ziphius cavirostris isolate SWFSC ID z0004472 mitochondrion, complete genome

ATGATCAACATTCGAAAAACACACCCACTAATAAAAATCATCAATAACGCATTCATTGACCTTCCCACTC

CATCAAACATCTCCTCATGATGAAACTTCGGCTCCTTACTCGGCCTCTGCCTCATCATACAAATTCTCAC

AGGCCTGTTCTTAGCAATACACTATACACCAGACACAACAACAGCCTTCTCATCTGTTGCACACATTTGC

CGAGACGTCAACTATGGCTGAATCATCCGATACCTACACGCAAACGGGGCCTCCATATTCTTTATCTGCC

TTTACGCCCATATCGGACGTGGACTATATTACGGCTCTTATATCTTTCAAGAAACATGAAACATCGGAGT

AATCCTACTCCTTGCAGTTATAGCCACCGCATTTGTGGGCTATGTCCTACCTTGAGGACAAATATCATTC

TGAGGTGCAACCGTCATCACAAATCTCCTATCCGCTATCCCCTATATCGGCACTACTCTCGTCGAATGAA

TCTGAGGTGGTTTTTCAGTAGATAAAGCCACACTAACACGCTTCTTTGCCTTCCATTTCATCCTTCCATT

TATTATTTTAGCCCTAGCAGCCGTCCACTTACTATTTCTCCATGAAACAGGATCTAATAACCCCACAGGA

ATCCCATCCGATATAGACAAAATCCCATTCCACCCTTATTACACAATCAAAGACATCCTAGGAGCCCTAC

TATTAATTGCAATCCTACTCGCACTAACCCTATTCGCACCCGACCTGCTAGGAGATCCCGATAACTATAC

CCCAGCAAATCCACTCAGCACCCCAGCACACATTAAACCAGAATGATATTTCCTATTCGCATACGCAATC

CTACGATCAATTCCTAATAAACTAGGAGGCGTATTAGCCCTACTCCTTTCAATCCTTGTCCTACTGTTTA

TTCCTCTACTCCACACGTCTAAACAACGAAGTATAATATTCCGACCCTTCAGTCAATTCCTCTTCTGATT

ATTAGTCGCAGACTTCCTAACCCTAACATGGATCGGAGGCCAACCCGTAGAACACCCCTACATGATCTTA

GGCCAACTAGCATCTATCTTATATTTTCTCCTAATCCTAGTACTAATACCAATAGCTAGCCTCATCGAGA

ATAAACTACTGAAATGAAGA

Short-finned pilot whale (*Globicephala macrorhynchus*)

>NC_019578.2:14199-15338 Globicephala macrorhynchus mitochondrion, complete genome

ATGACCAACATCCGAAAAACACACCCACTAATAAAAATCATCAATAACACATTCATTGACCTACCCACTC

CATCTAACATCTCCTCATGATGAAACTTTGGCTCCTTACTAGGCCTCTGCTTAATTATACAAATCCTAAC

AGGTTTATTCCTAGCAATACATTACACACCAGACACCTCAACCGCTTTTTCATCAGTCGCACACATCTGT

CGAGACGTCAACTATGGCTGATTCATCCGCTACCTACATGCAAACGGAGCTTCCATATTCTTCATCTGCC

TTTACGCCCACATCGGACGTGGCTTATACTATGGCTCTTATATATTCCAAGAAACATGAAACATTGGTGT

GCTCCTACTACTAACAGTCATGGCCACTGCATTCGTAGGCTATGTTCTACCCTGAGGACAGATATCATTC

TGAGGCGCAACCGTCATCACCAATCTCCTATCAGCAATCCCTTACATCGGCACCACCTTAGTAGAATGAA

TCTGAGGTGGATTTTCCGTAGACAAAGCAACACTAACACGTTTTTTCGCTTTCCACTTTATCCTCCCATT

CATCATCACAGCATTAGTAGCTGTCCACCTGCTATTCCTACACGAAACAGGATCCAATAACCCCATAGGA

ATCCCATCCAACATAGACATAATTCCATTCCACCCCTATTATACAATTAAAGACATCCTAGGCGCCCTAC

TCTTAATCCTAGCACTACTAACACTAACCCTATTCACCCCTGACCTACTAGGAGACCCTGATAACTATAC

TCCAGCAAATCCACTAAGCACCCCTGCACACATCAAACCAGAATGATATTTCCTATTCGCATATGCAATC

TTACGATCAATTCCCAATAAACTTGGAGGAGTACTAGCACTATTACTTTCCATCCTTATCCTAATCTTTA

TCCCAATACTTCAAACTTCCAAACAACGAAGTATAATATTCCGTCCCTTTAGCCAACTTTTATTCTGAAC

CCTAATCGCAGACCTCCTAACCTTAACATGAATTGGAGGCCAACCTGTAGAACACCCATACATCATTGTA

GGCCAATTAGCATCTATCTTATATTTCCTTCTAATCTTAGTGCTAATACCAACAGTCAGCCTTATTGAAA

ATAAACTTCTAAAATGAAGA
